# IgE-Mediated allergy to wheat in a child with celiac disease – a case report

**DOI:** 10.1186/1710-1492-10-56

**Published:** 2014-11-10

**Authors:** Tiffany Wong, Hin Hin Ko, Edmond S Chan

**Affiliations:** BC Children’s Hospital, Allergy Clinic, Room 1C31B, 4480 Oak St, Vancouver, BC V6H 3 V4 Canada; Department of Medicine, Division of Gastroenterology, 770-1190 Hornby Street, Vancouver, BC V6Z 2 K5 Canada

**Keywords:** Celiac disease, Allergy, Wheat

## Abstract

**Introduction:**

Celiac disease and immediate type hypersensitivity to wheat are immune responses with different pathogenic mechanisms. Both diseases are well known entities but their coexistence in the same patient is rarely reported. This is a unique case presentation of a patient with celiac disease who developed concomitant IgE-mediated wheat allergy and presented with immediate symptoms in two body systems.

**Case presentation:**

We report the case of a girl with celiac disease who subsequently developed IgE-mediated hypersensitivity to wheat. The patient is a Caucasian female who was diagnosed with celiac disease at 18 months of age after presenting with recurrent vomiting and failure to thrive. Her anti-tTG antibody level was greater than 200 E.U. and biopsy results from endoscopy were consistent with celiac disease. Specific IgE antibody to wheat was negative at 2 years of age. Around seven years of age, she developed immediate symptoms of urticaria, cough and shortness of breath with accidental exposures to wheat. Specific IgE antibody testing was repeated and positive to wheat (42.5 kU/L), as well as rye (33.9 kU/L), barley (53.4 kU/L) and oat (11.3 kU/L). At 9 years of age, skin prick testing was positive to wheat, barley and rye but negative to oat. The patient has subsequently tolerated an open oral food challenge to oat. She continues to avoid wheat, rye and barley and carries an epinephrine autoinjector at all times.

**Conclusion:**

To our knowledge, this is the first report of a patient with celiac disease and concomitant IgE-mediated allergy to wheat presenting with immediate symptoms in two body systems. Although the pathophysiology of these diseases is different, this case demonstrates that they are not exclusive of one another. In patients who develop unexplained symptoms consistent with IgE-mediated allergy, an allergy assessment should be considered.

## Background

Celiac disease and immediate type hypersensitivity to wheat are immune responses with different pathogenic mechanisms [1]. Both diseases are well known entities but their coexistence in the same patient is rarely reported. One patient from Spain has been reported to have likely celiac disease and positive skin prick testing to wheat, with immediate isolated gastrointestinal symptoms upon ingestion [2]. To the best of our knowledge, there have not been any cases reported in North America.

## Case presentation

At 18 months of age, a Caucasian female presented with persistent daily vomiting and failure to thrive. Complete blood count, liver function tests, viral serologies and serum amylase were normal. The anti-tissue transglutaminase antibody level was greater than 200 RU/mL (normal <20). During endoscopy, mild gastric antral inflammation and scalloping of the duodenal mucosa was seen. Biopsies of the gastric antrum showed chronic antritis and the duodenum showed villous atrophy and increased intraepithelial lymphocytes, consistent with celiac disease. She was placed on a gluten-free diet, although she had intermittent accidental ingestion of gluten with occasional vomiting. At 2 years of age, the specific IgE antibody to wheat was negative.

Around 7 years of age, there was a change in her symptoms whereby she immediately developed mouth tingling with accidental gluten ingestion. The tingling sensation lasted about ten minutes, and culminated in vomiting. There were no other associated symptoms, including respiratory distress, urticaria or angioedema. Anti-tissue transglutaminase antibody level was within normal limits (7.9 RU/mL) at this time. No further testing for anti-tissue transglutaminase has been done subsequent to this.

At 8 years of age, she attended a birthday party where some wheat flour was thrown into the air and came into contact with her skin. She immediately developed urticarial lesions on the areas exposed to wheat flour. She subsequently saw an allergist and was found to have positive skin tests to wheat, oat and rye. Specific-IgE levels were positive to wheat (42.5 kU/L), rye (33.9 kU/L), barley (11.3 kU/L) and oat (11.3 kU/L). An epinephrine autoinjector was prescribed.

Later that year, she was eating rice pasta which she had previously tolerated. She immediately developed coughing, shortness of breath, a tingly mouth and possible wheezing. Her symptoms resolved without use of epinephrine. She ate the rice pasta a subsequent time and developed immediate shortness of breath and pruritus over her chin. She ate home-made rice pasta on two further occasions and also developed shortness of breath and chin pruritus on both occasions. We suspect that the rice flour used was likely contaminated with wheat, as all other foods mixed with rice pasta were being tolerated regularly in her diet. She later tolerated a different batch of the same rice pasta brand, confirming that the previous batch she had reacted to multiple times had been contaminated with wheat. Subsequently, she accidentally ate pizza made with wheat flour and had immediate mouth tingling and vomiting. An oral challenge to wheat was contraindicated given this very recent history, as well as her need to avoid wheat due to celiac disease.

She was referred to our centre at 9 years of age for evaluation. Other than celiac disease and reactions to wheat, she was otherwise healthy and taking no medications. Parents denied the child had any history of drug allergy, asthma or angioedema. Physical examination showed a well appearing child with an unremarkable physical exam. Skin prick testing (Omega Laboratories Ltd, Montreal, Canada) was positive to wheat (8×7 mm), barley (5×6 mm) and rye (5×9 mm). Skin prick testing was negative to oat, and the patient tolerated an oral challenge to cooked pure oat. We recommended that she continue to eat oat, and ensure that it is not contaminated with gluten. Repeat anti-tTG antibody level at 10 years of age was normal (5.7 E.U). This illustrates that she generally is good at avoiding gluten, and the accidental exposures are not frequent for her. However, when they occur they result in severe, immediate reaction. (Note: There was a change in the assay used to measure anti-tTG antibody at our centre with a concomitant change in reported units).

Concomitant IgE-mediated allergy and celiac disease in individuals is rarely reported. We were able to identify only one case report of a child in Spain who had suspected celiac disease with symptoms of abdominal pain, gastric distension, increased flatulence and vomiting immediately after intake of food containing wheat and/or rye since 8 months of age [2]. She had elevated anti-tTG antibody levels and HLA-DQ2 haplotype but endoscopy was inconclusive as the patient was on a gluten-free diet at the time. The patient had positive skin prick tests to wheat, barley, rye, and oats, but passed open oral food challenges to corn flakes made of wheat, soy, oatmeal and rice. A subsequent single-blind, placebo controlled food challenge to wheat flour mixed with rice induced nausea, abdominal pain and vomiting 30 minutes after ingestion. We speculate that she did not react to the corn flakes because there may have not been a sufficient amount of wheat in the cereal to meet her threshold dose for reaction.

To our knowledge, this is the first report of a patient with biopsy confirmed celiac disease and concomitant IgE-mediated allergy to wheat presenting with immediate symptoms in two body systems outside of the gastrointestinal tract. This case is also unusual because this patient developed sensitization later in childhood (her initial specific IgE to wheat was negative), whereas most wheat allergic children are sensitized in infancy [3]. One group studied 57 children with celiac disease and found that four had positive specific IgE to wheat [4]. Clinical manifestations of immediate type hypersensitivity were not described in these children. Our patient had a recent, convincing history of immediate reaction to wheat. However, our case also illustrates the potential difficulty in obtaining consent for an oral food challenge to wheat in patients who might have a less convincing history of immediate reaction but must avoid wheat due to confirmed celiac disease.

Wheat consists of water-soluble albumins, gliadins, glutenins and salt-soluble globulins [5]. Specific allergens implicated in different immunologic diseases pertaining to wheat remain unclear. Albumins and globulins appear to be important proteins contributing to IgE-mediated hypersensitivity to wheat protein [6, 7]. Studies have identified 15 kd, 20 kd and 47 kg wheat proteins as potential allergens in children with confirmed IgE mediated allergy to wheat [7, 8]. In contrast, A2-gliadin, a 33 amino acid peptide, is thought to be the immunologic target in celiac disease [9]. Omega 5 gliadin and high molecular weight glutenins are implicated allergens in wheat dependent exercise induced anaphylaxis [2, 9]. Omega 5 gliadin specific IgE component testing studies have reported 80% sensitivity, positive predictive value of 37.5% and negative predictive value of 91% [10, 11]. However, specific allergens implicated in IgE-mediated hypersensitivity to wheat need to be further elucidated before component testing with specific IgE antibodies can be pursued. For anaphylaxis to wheat without exercise, published reports for specific allergens have been contradictory [12–14].

Repeat skin and serum specific IgE testing over time in this patient has strength in that it allows us to determine that IgE-mediated allergy developed over time in this patient, instead of being pre-existing. However, the laboratory work up in this patient has limitations. Pursuing further specific IgE antibodies to specific components of wheat would be of academic interest; however, these tests are not available to us in our region. In addition, the specific component allergens implicated in IgE-mediated wheat allergy have not been fully elucidated, as outlined above. Further testing for wheat protein components was not pursued as results would have unclear significance and would not have changed clinical management.

We speculate it might be possible that our patient developed IgE-mediated wheat allergy as a consequence of lack of regular oral exposure to wheat. Regular oral exposure to a food is currently thought to result in maintenance of oral tolerance to the food, whereas lack of oral exposure may augment the risk of IgE-mediated sensitization [15]. Strict avoidance of gluten is recommended for all patients with celiac disease, which should theoretically increase the risk of IgE-mediated wheat allergy in patients with celiac disease beyond the numbers described in the literature. One possibility for why immediate hypersensitivity to wheat is not seen more often is a lack of recognition of IgE-mediated wheat allergy as an explanation for idiopathic symptoms (e.g. urticaria) in patients with celiac disease, i.e. symptoms not recognized as accidental ingestion of products contaminated with wheat.

## Conclusion

Although the pathophysiology of celiac disease and immediate type hypersensitivity to wheat differs, this case demonstrates that the diseases are not exclusive of one another. In patients "with celiac disease: who develop unexplained symptoms consistent with IgE-mediated allergy, an allergy assessment should be considered.

## Consent

Written informed consent was obtained from the patient for publication of this Case report and any accompanying images. A copy of the written consent is available for review by the Editor-in-Chief of this journal.

## Authors’ information

TW is a Clinical Immunology and Allergy subspecialty resident at the BC Children’s Hospital, Department of Pediatrics, Division of Allergy and Immunology.

HHK is a Clinical Assistant Professor at the University of British Columbia, Division of Gastroenterology, MD, FRCPC.

ESC is a Clinical Associate Professor and the Division Head of Allergy & Immunology, Department of Pediatrics at the BC Children’s Hospital.

## References

[1] Sapone A, Bai JC, Ciacci C, Dolinsek J, Green PH, Hadjivassiliou M, Kaukinen K, Rostami K, Sanders DS, Schumann M, Ullrich R, Villalta D, Volta U, Catassi C, Fasano A (2012). Spectrum of gluten-related disorders: consensus on new nomenclature and classification. BMC Med.

[2] Torres JA, Sastre J, Heras MD, Cuesta J, Lombardero M, Ledesma A (2008). IgE-mediated cereal allergy and latent celiac disease. J Invest Allerg Clin.

[3] Sampson HA (1989). Food allergy. J Allergy Clin Immunol.

[4] Armentia A, Arranz E, Hernandez N, Garrote A, Panzani R, Blanco A (2008). Allergy after inhalation and ingestion of cereals involve different allergens in allergic and celiac disease. Recent Pat Inflamm Allergy Drug Discov.

[5] Björkstén F, Backman A, Järvinen KA, Lehti H, Savilahti E, Syvänen P, Kärkkäinen T (1977). Immunoglobulin E specific to wheat and rye flour proteins. Clin Allergy.

[6] Baldo BA, Wrigley CW (1978). IgE antibodies to wheat flour components. Studies with sera from subjects with baker's asthma or coeliac condition. Clin Allergy.

[7] Jones SM, Magnolfi CF, Cooke SK, Sampson HA (1995). Immunologic cross-reactivity among cereal grains and grasses in children with food hypersensitivity. J Allergy Clin Immunol.

[8] James JM, Sixbey JP, Helm RM, Bannon GA, Burks AW (1997). Wheat alpha-amylase inhibitor: a second route of allergic sensitization. J Allergy Clin Immunol.

[9] Shan L, Molberg Ø, Parrot I, Hausch F, Filiz F, Gray GM, Sollid LM, Khosla C (2002). Structural basis for gluten intolerance in celiac sprue. Science.

[10] Jacquenet S, Morisset M, Battais F, Denery-Papini S, Croizier A, Baudouin E, Bihain B, Moneret-Vautrin DA (2009). Interest of ImmunoCAP system to recombinant omega-5 gliadin for the diagnosis of exercise-induced wheat allergy. Int Arch Allergy Immunol.

[11] Matsuo H, Dahlström J, Tanaka A, Kohno K, Takahashi H, Furumura M, Morita E (2008). Sensitivity and specificity of recombinant omega-5 gliadin-specific IgE measurement for the diagnosis of wheat-dependent exercise-induced anaphylaxis. Allergy.

[12] Baar A, Pahr S, Constantin C, Giavi S, Manoussaki A, Papadopoulos NG, Ebner C, Mari A, Vrtala S, Valenta R (2014). Specific IgE reactivity to Tri a 36 in children with wheat food allergy. J Allergy Clin Immunol.

[13] Shibata R, Nishima S, Tanaka A, Borres MP, Morita E (2011). Usefulness of specific IgE antibodies to omega-5 gliadin in the diagnosis and follow-up of Japanese children with wheat allergy. Ann Allergy Asthma Immunol.

[14] Constantin C, Quirce S, Poorafshar M, Touraev A, Niggemann B, Mari A, Ebner C, Akerström H, Heberle-Bors E, Nystrand M, Valenta R (2009). Micro-arrayed wheat seed and grass pollen allergens for component-resolved diagnosis. Allergy.

[15] Lack G (2012). Update on risk factors for food allergy. J Allergy Clin Immunol.

